# Prebiotic chemistry in neutral/reduced-alkaline gas-liquid interfaces

**DOI:** 10.1038/s41598-018-36579-7

**Published:** 2019-02-13

**Authors:** Cristina Mompeán, Margarita R. Marín-Yaseli, Patricia Espigares, Elena González-Toril, María-Paz Zorzano, Marta Ruiz-Bermejo

**Affiliations:** 1 Department of Molecular Evolution, Centro de Astrobiología (CSIC-INTA), Carretera de Ajalvir km 4, Torrejón de Ardoz, 28850 Madrid, Spain; 2 Department of Planetology and Habitability, Centro de Astrobiología (CSIC-INTA), Carretera de Ajalvir km 4, Torrejón de Ardoz, 28850 Madrid, Spain; 3 Atmospheric Sciences Group, Department of Computer Sciences, Electrical and SpaceEngineering, Space Division, Luleå University of Technology, Luleå, Sweden

## Abstract

The conditions for the potential abiotic formation of organic compounds from inorganic precursors have great implications for our understanding of the origin of life on Earth and for its possible detection in other environments of the Solar System. It is known that aerosol-interfaces are effective at enhancing prebiotic chemical reactions, but the roles of salinity and pH have been poorly investigated to date. Here, we experimentally demonstrate the uniqueness of alkaline aerosols as prebiotic reactors that produce an undifferentiated accumulation of a variety of multi-carbon biomolecules resulting from high-energy processes (in our case, electrical discharges). Using simulation experiments, we demonstrate that the detection of important biomolecules in *tholins* increases when plausible and particular local planetary environmental conditions are simulated. A greater diversity in amino acids, carboxylic acids, N-heterocycles, and ketoacids, such as glyoxylic and pyruvic acid, was identified in *tholins* synthetized from reduced and neutral atmospheres in the presence of alkaline aqueous aerosols than that from the same atmospheres but using neutral or acidic aqueous aerosols.

## Introduction

*Tholins* are complex organic materials obtained by activation, using several energy sources, of atmospheres that contain CO, CO_2_ or CH_4_ as carbon sources and N_2_ or NH_3_ as nitrogen sources. The syntheses of *tholins* is of high interest in the field of the prebiotic chemistry and in studies about the origin of life because in their production are simulated plausible prebiotic conditions that potentially may lead to the production of biochemically interesting organic molecules. The purpose of these simulation experiments is to obtain a better understanding of the likely environments in which life could have emerged and the first steps of a primitive biology via accumulation of multi-carbon biomolecules. Miller’s experiment^1^ is famous due to the production of *tholins*. Since this successful experiment, most of the recent Miller-type experiments simulating earth conditions used water in vapour form (e.g.^2^) or liquid water (e.g.^3–5^). A few examples reported using water in the solid state (e.g.^6^), and interestingly, in the last decade, the role of the aqueous aerosols in these types of experiments is being explored^7–9^. The possible importance of aerosols in the origin of life on the early Earth has been emphasized in recent years. Aqueous aerosols are considered to be “prebiotic microreactors”^10,11^ and exhibit an efficient variation in the reactivity of systems^12,13^. Aqueous aerosols can enhance the yield of polar organic compounds^8,14,15^, improve the formation of determinate organics against others^15^ and positively influence non-enzymatic polymerization reactions^16,17^.

On the other hand, pure water was employed in the majority of Miller-type experiments, and the roles of salinity and pH have been rarely studied in this type of experiment^9,18–21^. Because salinity and pH are important physico-chemical parameters on a planetary scale and in local environments and since the right environment (in the case that this one was unique) for the emergence of life currently remains unknown, both parameters should be taken into account in Miller-type experiments. Indeed, the formation of an aerosol depends only on a liquid water-air interface and a physical mechanism that ejects bubbles into the atmosphere, such as wind, sea waves or shock waves^22,23^. These water surfaces can correspond to oceans, internal seas, lakes or rives and so on, which can in turn present different compositions in terms of salinity and pH. The introduction of these parameters together with others that are more widely studied, such as the compositions of the gas mixtures, might lead to finding new clues about the puzzling trouble of the origin of life.

Therefore, in the present work, we investigate the role of the pH in the presence of aqueous aerosols in Miller-type experiments to understand what conditions are more favourable to the accumulation of organics, which may be the main characters in a plausible emergence of a primitive biology. Thus, a CH_4_ + N_2_ + H_2_ atmosphere or a more reducing atmosphere composed by CH_4_ + NH_3_ + H_2_, which can simulate the composition of volcanic gas mixtures, was activated by spark discharges in the presence of aqueous aerosols produced using several solutions with different initial pH values. The choice of these gas mixtures constraints the simulations to a particular planetary local environment, volcanoes next to any water surface. The complex mixtures obtained were studied via elemental analysis, FT-IR spectroscopy and GC-MS. Table 1 summarizes the plausible prebiotic synthetic conditions explored herein. Note that experiment **6** can seem very similar to that reported previously by Johnson *et al*.^7^. However, the conditions and the techniques for the production of aerosols are truly different, as are the prebiotic conditions simulated. Johnson *et al*.^7^ simulated the steam vapour that it is formed in volcanic eruptions and the subsequent formation of aerosols, whereas in the present case, the aerosols are formed from a pool of liquid water at r.t. that simulates any liquid water surface. The analytical techniques and the conclusions of the study by Johnson *et al*.^7^ and the work present here are also different. In the present work, a wide screening for polar organic molecules was performed via GC-MS, whereas in the case of Johnson *et al*.^7^, HPLC was used as analytical technique, and amino acids were mainly reported, although in both cases, the role of the aerosols was revealed.

Herein, as expected, a set of amino acids, carboxylic acids and N-heterocycles were identified in the hydrophilic *tholins* by GC-MS, but precursors of sugars were also detected. Moreover, ketoacids, such as glyoxylic acid and pyruvic acid, were identified for the first time in this type of experiment. Our analysis confirms that alkaline aerosol environments, compared to acidic or neutral environments, are particularly effective microreactors that favour the emergence of a wealth of compounds likely implicated in the first steps of a primitive biochemistry.

## Materials and Methods

### Simulation of alkaline reductive environments

The syntheses of the hydrophilic and hydrophobic *tholins* via spark discharges experiments in an atmosphere of CH_4_ + N_2_ + H_2_ and liquid water, aqueous aerosols or saline aqueous aerosols (experiments **1**–**4**, Table 1) are described in references^8^ and^9^. New experiments were carried out following the methodology previously developed using a gas mixture CH_4_:NH_3_:H_2_ (40:30:30) purchased from Air Liquid (experiments **5**–**8**, Table 1. For details, see the Supporting Information).

**Table 1:** Miller-type experiments. Synthetic conditions explored in this study.

Experiment	Atmosphere	Salts	Initial pH	Aerosol	Ref.
1	CH_4_ + N_2_ + H_2_	−	7	−	This work and^8^
2	CH_4_ + N_2_ + H_2_	−	7	+	This work and^8^
3	CH_4_ + N_2_ + H_2_	+	9.8	+	This work and^9^
4	CH_4_ + N_2_ + H_2_	+	12	+	This work and^9^
5	CH_4_ + NH_3_ + H_2_	−	7	−	This work
6	CH_4_ + NH_3_ + H_2_	−	7	+	This work
7	CH_4_ + NH_3_ + H_2_	+	9.8	+	This work
8	CH_4_ + NH_3_ + H_2_	+	12	+	This work
9	CH_4_+N_2_+H_2_	+	4.8	+	This work and^9^
10	CH_4_+N_2_+H_2_	+	5.8	+	This work and^18^
11	CH_4_+N_2_+H_2_	+	7.8	+	This work and^9^

The gas mixtures were active for 3 days in the presence of pure water (−) or saline solutions (+) as well as without (−) or with an active aerosol cycle (+). The temperature was constant throughout the reaction time, 38 °C.

### Instrumental Analyses

#### Gas Chromatography-Mass spectrometry (GC-MS)

GC-MS analyses in the full-scan mode were carried out on a 6850 network GC system coupled to a 5975 VL MSD with a triple-axis detector operating in electronic impact (EI) mode at 70 eV (Agilent) using an HP-5 MS column (crossbond 5% diphenyl-95% dimethyl polysiloxane, 30 m ×0.25 mm i.d. ×0.25 µm film thickness) and He as the carrier gas.

### Analytical procedure

In all experiments, we collected a solution and an insoluble solid. These were separated by centrifugation and immediately freeze-dried using a standard lyophilizer; in this way, we obtained a hydrophilic *tholin* and a hydrophobic *tholin*. All samples were stored at −20 °C under a nitrogen atmosphere until they were analysed. In the case of the experiments in which saline solutions were used, prior to GC-MS analysis of the organics, the hydrophilic *tholins* were desalted by ion exchange chromatography [Dowex 50 W X 8–400 (H^+^)] using 5 N NH_4_OH as the first eluant (F_NH3_ fractions) and water as the second eluant (F_H2O_ fractions). The fractions were lyophilized and subsequently weighed. For the *identification of polar organic molecules* in all freeze-dried fractions: (i) The samples were hydrolysed with 6 M HCl at 110 °C for 24 h and then freeze dried to remove water, HCl and any volatile organics; (ii) two milligrams of each hydrolysed sample in 75 µL of BSTFA with 1% TMCS [N,O-bis(trimethylsilyl)trifluoroacetamide with trimethylchlorosilane, from Thermo Scientific] was heated at 70 °C for 19 h to obtain the respective TMS derivatives; and (iii) the derivatized samples were analysed by GC-MS using the following GC oven program: 60 °C (initial temperature) with a hold time of 1.5 min, heating to 130 °C at 5 °C/min with a hold time of 11 min, heating to 180 °C at 10 °C/min with a hold time of 10 min and heating to 220 °C/min at 20 °C/min with a final hold time of 15 min. One microlitre of each sample was injected. The temperature of the injector was 275 °C, and the injections were performed in splitless mode. The detector temperature was 300 °C. The flow rate was 1.1 mL/min. As a rule, identification of the GC-MS peaks attributed to organic compounds was verified by comparison with the retention times and mass spectra of external standards (purchased from Sigma-Aldrich and Fluka).

### Quantification of α-ketoacids

In the particular case of glyoxylic acid (**c16**) and pyruvic acid (**c17**), a first quantification was achieved using the multiple point external standard method, using standard solutions from 10 ppm to 100 ppm. The following procedure was used to prepare their corresponding oxime-TMS derivatives standards: (i) The needed milligrams of the α-ketoacids **c16** and **c17** for each standard solution were heated at 60 °C for 30 min in 500 µL of a hydroxylamine hydrochloride solution at pH 12 (20 mg of hydroxylamine hydrochloride in 1 mL of NaOH 2 N); (ii) 200 µL of HCl 6 N was added; (iii) the final mixtures were extracted with 500 µL of ethyl acetate (x2) and 500 µL diethyl ether (x1); (iv) the organic layers were combined and dried under a continuous flow of N_2_; (v) the samples were freeze-dried to remove residual water; and (vi) the dried residues were heated at 80 °C for 3 h in 100 µL of BSTFA + 1% TMCS. The oxime-TMS derivatives were injected and analysed by GC-MS as indicated above.

### Fractionation of the hydrophilic tholins

#### Concentration by ultrafiltration

The fractions obtained after ion exchange chromatography using 5 N NH_4_OH (F_NH3_ fractions) were subfractionated using Nanosep^®^ centrifugal devices (Pall, Life Sciences) to retain molecules above 3 kDa. Both the light fractions (<3 kD) and heavy fractions (<3 kDa) were analysed by polar organics as mentioned above.

### Multivariate analysis

Multivariate analysis (for dependent and independent factors) was carried out using a combination of constrained and unconstrained multivariate statistical methods to account for both the total variation in the data and the variation explained by the environmental data. This statistical tool is very useful for comparing results of different experiments in which different variables have been applied. Of all the elements analysed, we retained 8 variables that had no missing values: milligrams of hydrophilic *tholin* (WSOM, weight of soluble organic matter), milligrams of hydrophobic *tholin* (WIOM, weight of insoluble organic matter), final pH, number of amino acids identified, number of carboxylic acids identified, number of N-heterocycles identified, number of polyols identified and total number of organic compounds identified. An absence-presence matrix with experimental conditions (absence-presence of NH_3_ in the reduced atmosphere used, salts, and aerosol) and the initial pH was generated. Detrended correspondence analysis (DCA) was performed to determine the modality of the sequence data. The analysis resulted in 0.612 segment lengths for the variable data and 1.879 segment lengths for the experimental conditions matrix. Both values were less than 3, indicating that the linear and unimodal models could be used (redundancy analysis or canonical correlation analysis). Finally, a redundancy analysis was performed because allows explain as much variance as possible and studying the influence of the different variables and experimental conditions. The significance of the first axis and that of all axes combined were tested using Monte Carlo permutation tests. DCA and RDA tests were performed using the multivariate data analysis software CANOCO 4.5 (Microcomputer Power, Ithaca, NY, USA)^24^. The program CANODRAW4.0 (in the CANOCO package) was used for graphical presentations.

## Results

### Syntheses and characterization of tholins

**Figure 1:**
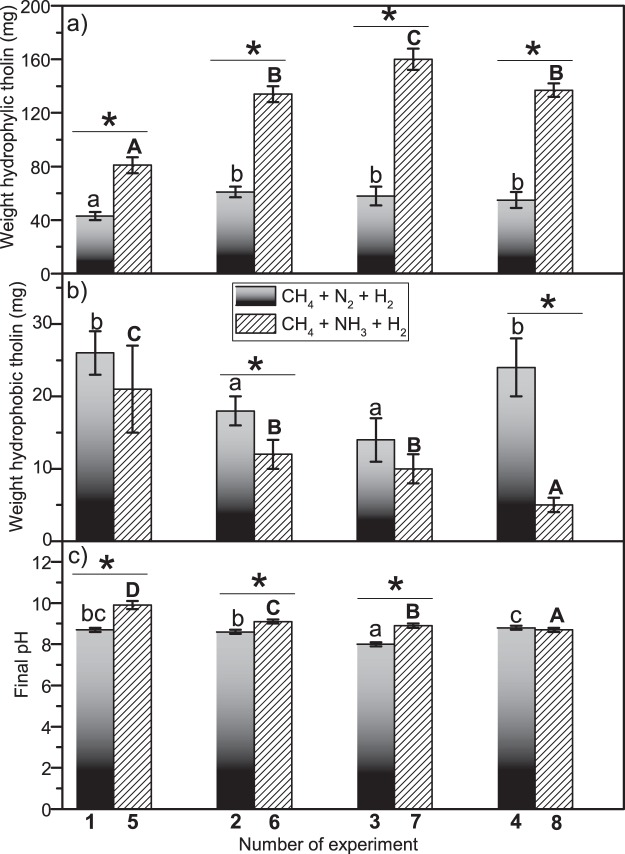
Histograms showing the effects of the aqueous aerosols and initial pH on (**a**) the final weight of the hydrophilic *tholins* (soluble organic matter); (**b**) the final weight of the hydrophobic *tholins* (insoluble organic matter); and (**c**) the final pH of the crude reactions. The number shown on the x-axis corresponds to the experiment number indicated in Table 1. Statistical test: pairs test based on the confidence interval comparison (α = 0.047). *Indicates differences within groups, and letters indicate differences between groups (CH_4_ + N_2_ + H_2_ group = lowercase letters; CH_4_ + NH_3_ + H_2_ group = capital letters). The average values and the standard deviations were calculated from the results of at least five experiments.

Hydrophilic *tholins* and hydrophobic *tholins* were obtained, either in experiments using an atmosphere of CH_4_ + N_2_ + H_2_ or an atmosphere of CH_4_ + NH_3_ + H_2_. Figure 1a shows the amount of hydrophilic *tholin* formed in each experiment. It is clear that the presence of NH_3_ improved the amount of soluble organic materials produced in all cases studied, and it was expected that the presence of aqueous aerosols increased the amount of soluble materials. By contrast, the presence of salts and pH variations did not have a strong influence on the total amount of soluble organic material fixed as hydrophilic *tholins*. In the case of the hydrophobic *tholins*, the effects observed were the opposite of those observed for the hydrophilic *tholins*. In general, the amount of hydrophobic *tholins* produced was less when CH_4_ + NH_3_ + H_2_ was used than when N_2_ was present in the simulations experiments, and a light influence of the initial higher pH values was observed in relationship to the weight of the hydrophobic *tholins* (Fig. 1b). On the other hand, in all cases, the final pH of these simulation experiments was dependent on the initial pH and salinity conditions and the presence of aqueous aerosols (Fig. 1c). In addition, the final pH depended on the atmosphere used. Higher final pH values were found for the syntheses carried out using a CH_4_ + NH_3_ + H_2_ atmosphere.

In summary, in a general way, NH_3_ improves the production of hydrophilic *tholins*, prevents the formation of hydrophobic *tholins* and increases the final pH of the crude reactions with respect to the simulation spark experiments that used N_2_ as a nitrogen source. Additionally, NH_3_ leads to a greater number of functional groups containing nitrogen and oxygen both in hydrophilic *tholins* and in hydrophobic *tholins*. Moreover, the salinity and pH significantly change the nature of both hydrophilic and hydrophobic *tholins* having a greater effect than the source of nitrogen (please see the Supplementary Information for the structural characterization of *tholins* by elemental analysis and FT-IR spectroscopy).

### GC-MS analyses of the hydrophilic tholins

**Figure 2:**
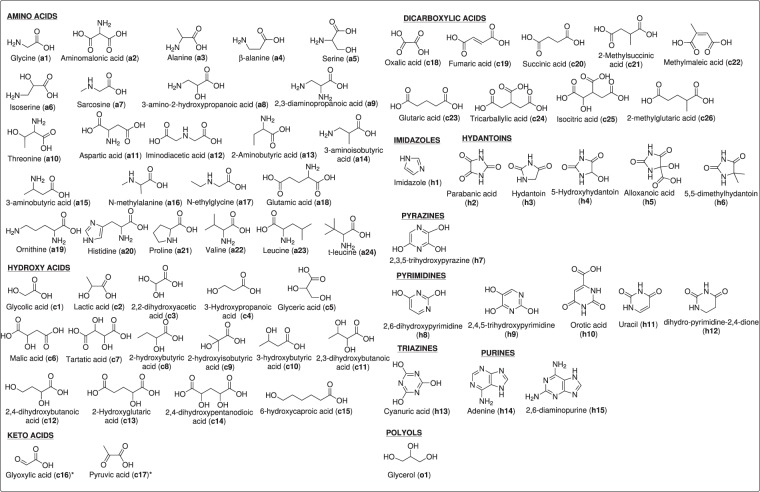
Polar organic compounds identified as their TMS derivatives by GC-MS in the present work. *Identified as oxime-TMS derivatives.

The hydrophilic *tholins* were analysed by GC-MS using BSTFA as a derivatization reagent to obtain the respective TMS derivatives of polar compounds, such as amino acids, carboxylic acids and several N-heterocycles. This derivatization method is not specific for each type of compound mentioned, but for comparative purposes, it provides an excellent general overview of the polar molecules present in all *tholins* synthesized. This analytical methodology allows discrimination among the various synthesis conditions tested to determine which are the most favourable from the point of view of the prebiotic production of polar bioorganics^12^. Figure 2 shows all of the analytes identified in this work.

#### Analyses of hydrophilic tholins from pure water experiments and F_NH3_ fractions

**Figure 3:**
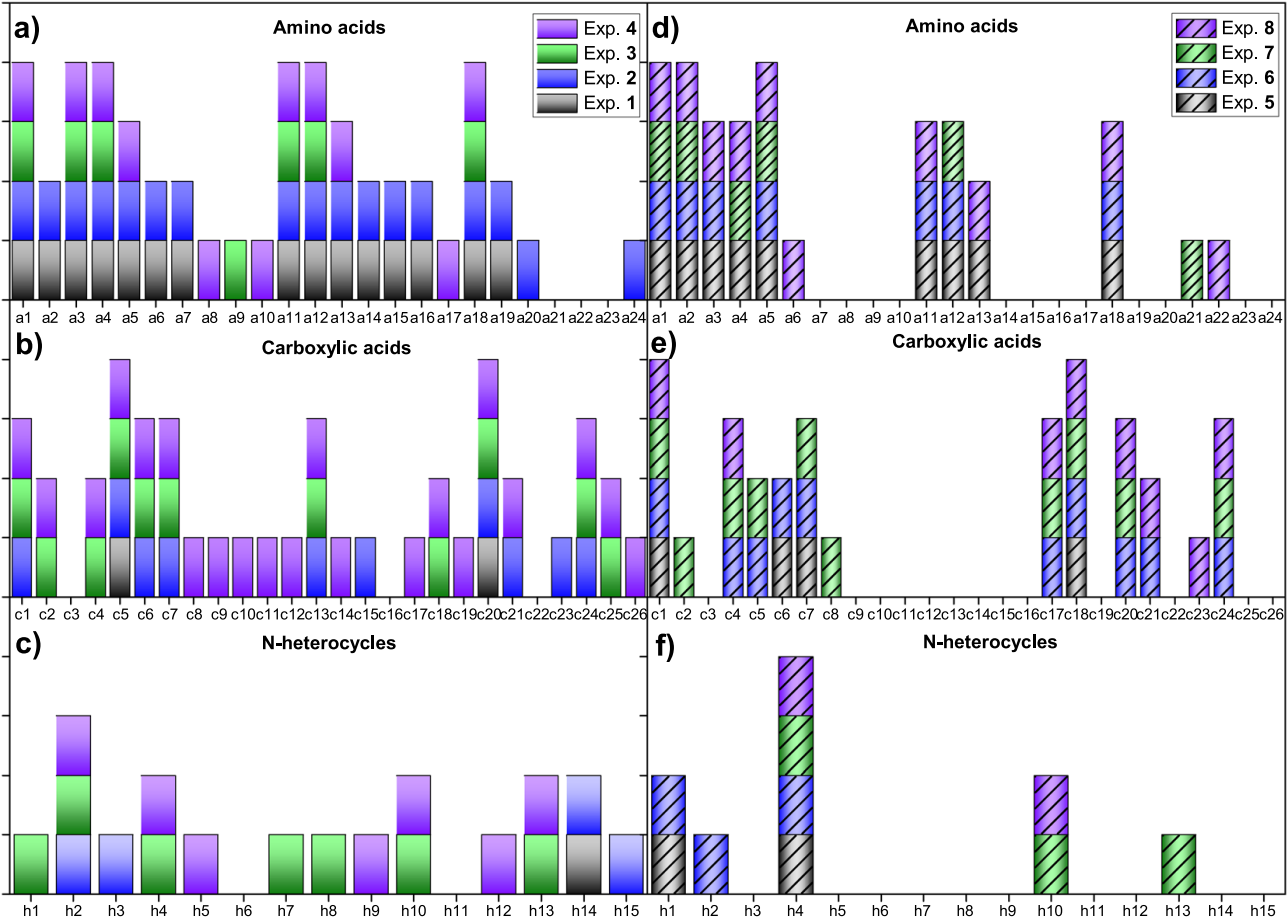
Organic polar compounds identified as their TMS-derivatives by GC-MS in acid hydrolysed hydrophilic *tholins* synthesized using pure water (experiments **1**–**2** and **5**–**6**) or in acid hydrolysed F_NH3_ fractions, which were obtained by desalting the hydrophilic *tholins* that were synthesized in the presence of alkaline aqueous aerosols at initial pH values of 9.8 or 12 (experiments **3**–**4** and **7**–**8**): (**a**,**c**,**d**) correspond to the amino acids, carboxylic acids and N-heterocycles, respectively, found in hydrophilic *tholins* and F_NH3_ fractions from experiments using an atmosphere of CH_4_ + N_2_ + H_2_; (**c**,**d**,**f**) correspond to amino acids, carboxylic acids and N-heterocycles, respectively, found in hydrophilic *tholins* and F_NH3_ fractions from experiments using an atmosphere of CH_4_ + NH_3_ + H_2_. The numbers under the abscissa axis correspond to the numeration of analytes shown in Fig. 2. The coloured bars indicate the presence of a concrete analyte in the corresponding sample.

In the case of the spark discharge experiments using saline solutions or alkaline aqueous aerosols, a previous step of desalting is necessary before GC-MS analyses of the hydrophilic *tholins*. Thus, those were desalted by an ion exchange resin, and a F_NH3_ fraction and a F_H2O_ fraction were obtained for experiments **3** and **4** according to the solvents used as eluants^9^. The relationship F_N3H_/F_H2O_ (mg of freeze-dried ammoniacal fraction/mg of freeze-dried water fraction) was 0.75 and 1.08 for experiments **3** and **4**, respectively. By contrast, the amount of F_H2O_ fraction was not significant for experiment **7**, and in experiment **8**, the relationship F_NH3_/F_H2O_ was 76. The F_NH3_ fractions from experiments **3**–**4** and **7**–**8** and the bulk hydrophilic *tholins* from experiments **1**–**2** and **5**–**6** were acid hydrolysed and analysed by GC-MS to determine the presence of organic polar compounds (Fig. 3). The comparison between the bulk hydrophilic *tholins* and F_NH3_ fractions was made because these fractions were predominant according to weight in the CH_4_ + NH_3_ + H_2_ experiments, and in the case of experiment **7**, the amount of the F_H2O_ fraction obtained was so small that it did not allow GC-MS analysis. The diversity in polar organics was greater for the CH_4_ + N_2_ + H_2_ series (Fig. 3a,c,e) than for the CH_4_ + NH_3_ + H_2_ series (Fig. 3b,d,f), especially when alkaline aqueous aerosols were used at an initial pH of 12. It is interesting that some carboxylic acids that are part of the reductive tricarboxylic acid cycle (rTCA), such as maleic acid (**c6**), pyruvic acid (**c17**), fumaric acid (**c19**), succinic acid (**c20**) and isocitric acid (**c25**), were identified in the F_NH3_ fraction synthesized under those experimental conditions (Fig. 3c, exp. **4**). Pyruvic acid (**c17**) was only identified as its oxime-TMS derivative in the CH_4_ + N_2_ + H_2_ series in experiment **4**; by contrast, for the CH_4_ + NH_3_ + H_2_ series, this compound was identified under any condition when aqueous aerosols were present (Fig. 3d, experiments **6**, **7** and **8**). This is the first time that an α-ketoacid that forms part of the rTCA has been detected in *tholins* from spark discharges experiments. The formation of the oxime-TMS derivative could be explained in the same way that the oxime-TMS derivative for the glyoxylic acid (**c16**) from HCN polymers forms^12^. The quantification of pyruvic acid (**c17**) shows yields based on the carbon-fixed levels of 0.00015% (0.037 µmoles), 0.003% (0.07 µmoles), 0.014% (0.36 µmoles) and 0.028% (0.71 µmoles) for experiments **4**, **6**, **7** and **8**, respectively. Note that the yields increase with the increase of the initial pH for the experiments of the CH_4_ + NH_3_ + H_2_ series and that the yield for the experiments at the initial pH 12 is greater when NH_3_ is used as a nitrogen source. The identification of tricarballylic acid (**c24**) (Fig. 3b,e) is also notable, and it is only identified in *tholins* when aqueous aerosols are used in their syntheses.

Note that although a greater diversity in the amino acids, carboxylic acids and N-heterocycles was observed in experiment **4**, the yields of the compounds were greater in experiment **8** than in experiment **4** (please see Table S1 in the Supplementary Information). Thus, in experiment **4**, a greater number of compounds was identified, but the yield in biomonomers was greater in experiment **8**.

#### Analyses of the FH2O fractions

**Figure 4:**
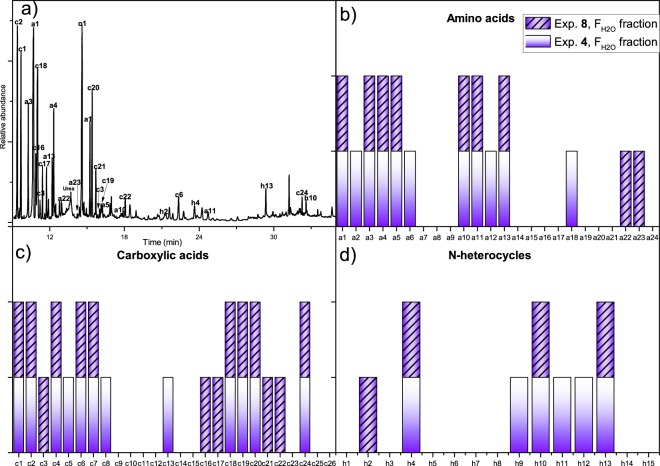
Organic polar compounds identified as their TMS derivatives by GC-MS in acid hydrolysed F_H2O_ fractions, which were obtained by desalting hydrophilic *tholins* synthesized in the presence of alkaline aqueous aerosols at an initial pH = 12 (experiments **4** and **8**): (**a**) Representative GC-MS chromatogram of F_H2O_ fraction from experiment **8**; (**b**–**d**) correspond to amino acids, carboxylic acids and N-heterocycles, respectively, found in F_H2O_ fractions from experiments **4** and **8**. The numbers shown on the peaks of the GC-MS chromatogram and under the abscissa axis correspond with the numeration of analytes shown in Fig. 2. The coloured bars indicate the presence of a concrete analyte in the corresponding sample.

The F_H2O_ fraction from experiment **8** was analysed and compared with the previous results from experiment **4** (Fig. 4). It is interesting for the further development of the “glyoxylic acid scenario” proposed by Eschenmoser^25^ that glyoxylic acid (**c16**) was identified as its oxime-TMS derivative in this fraction from experiment **8**. The yield of glyoxylic acid (**c16**) was 0.078% (2.98 µmol) based on the initial carbon input into the system. Additionally, pyruvic acid (**c17**) was detected with a yield for this fraction of 0.016% (0.41 µmoles). Together with the amount found in the F_NH3_ fraction, this result indicates a total amount of pyruvic acid from experiment **8** of 1.12 µmoles. It is also notable that glycerol (**o1**) (Fig. 4a) has a yield of 0.238% (6.23 µmoles). To our knowledge, this is the first time that a sugar derivative has been found in this type of prebiotic simulation.

The results shown in Fig. 4 indicate that the diversities of biomonomers and related compounds in experiments **4** and **8** are similar. This result is apparently not in agreement with the results discussed above. Thus, this point will be discussed later.

#### Analyses of non-hydrolysed samples and the subfractionations of the F_NH3_ fractions

For comparative purposes with the GC-MS analysis data discussed above and to develop a more exhaustive understanding of the system, non-hydrolysed samples were also studied. Moreover, the F_NH3_ fractions from experiments **7** and **8** were subfractionated by ultrafiltration using centrifugal filter devices with a cut-off of 3 kDa, collected in both cases from a light fraction (<3 kDa) and a heavy fraction (>3 kDa). These heavy fractions were 6% and 9% in weight of the total amount of hydrophilic *tholins* from experiments **7** and **8**, respectively. Either the light fractions or heavy fractions were acid hydrolysed and analysed by GC-MS. Figure S5 (please see the Supporting Information) shows the qualitative data analysis of the samples indicated in this section.

As a result, as expected, the diversity of amino acids and carboxylic acids is increased when the samples are hydrolysed, while the diversity in hydantoins is greater in the non-hydrolysed samples because this type of compound is partially or fully ring-opened under heating in an acid medium to lead to amino acids (Fig. S5a, S5b and S5c). On the other hand, in general, the number of organic compounds identified when F_NH3_ fractions **7** and **8** are subfractionated using ultrafiltration is greater than of the corresponding non-subfractionated samples (please compare Fig. 3b,d,f with Fig. S5d, S5e,S5f). Subfractionation allows the identification of compounds previously not found in the bulk F_NH3_ fractions.

At this point, it is difficult to compare the analytical results from the different experiments. On the one hand, one needs to take into account the experimental conditions of synthesis, and on the other hand, it is also important to note how the samples are handled because the number of organics identified depends on both aspects. To provide a global view, a statistical multivariate analysis was carried out, considering all of the organics identified in each sample and the experimental synthetic conditions.

### Multivariate analysis

**Figure 5:**
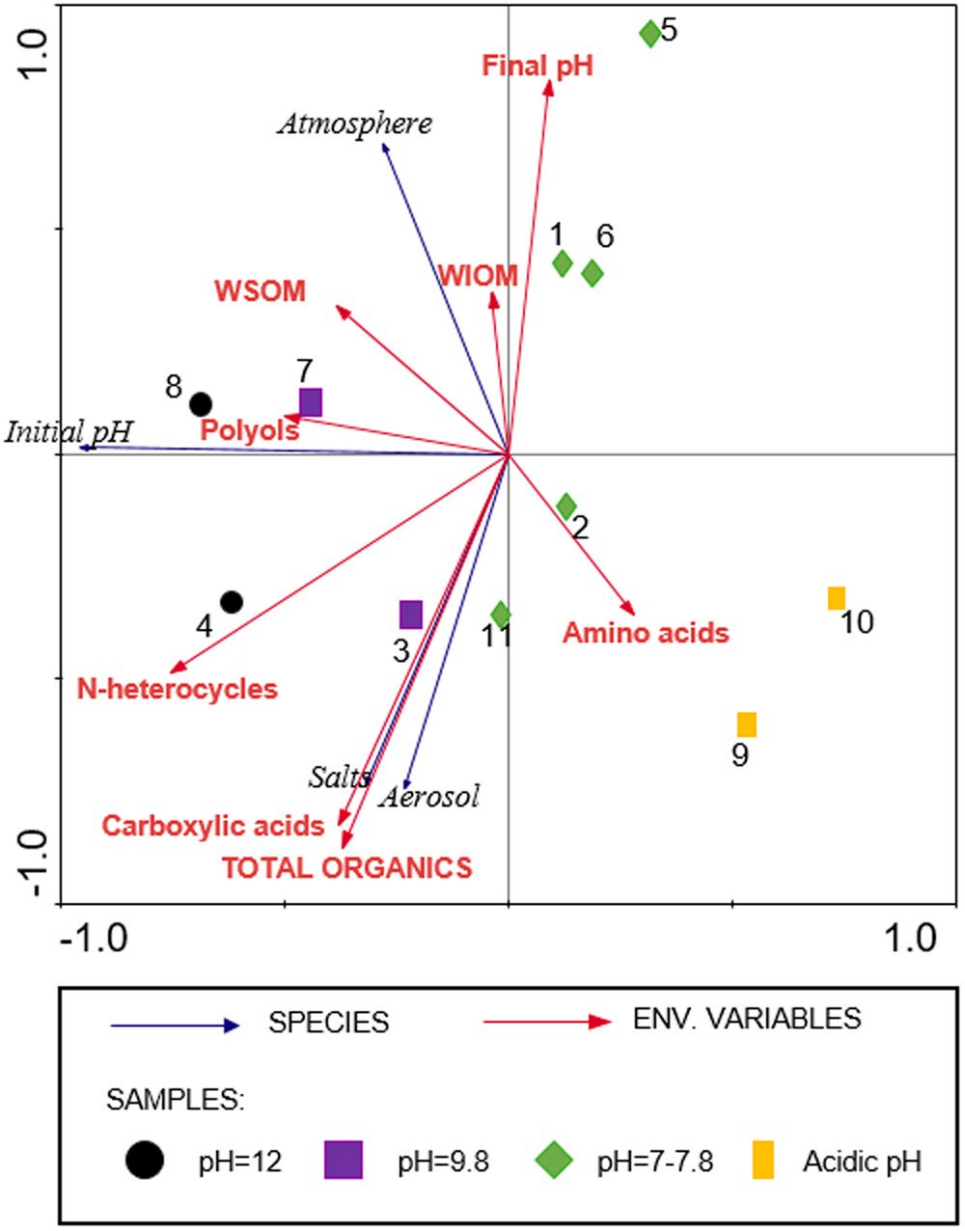
RDA triplot correspondence analysis relating variables and experimental conditions. Experimental conditions are shown by blue arrows. Variables used in the analysis are shown by red arrows. Experiments are indicated by numbers and different symbols depending on the initial pH. The synthetic conditions of experiments **1**–**8** are shown in Table 1. The initial pH of experiments **9**, **10** and **11** was 4, 5.8 and 7.8, respectively, and an atmosphere of CH_4_ + N_2_ + H_2_ (40:30:30), saline aqueous aerosols and spark discharges were used in the same way as experiments **3** and **4** (Table 1).

The RDA technique generates an ordination diagram in which axes are created by a combination of variables^26^. The eigenvalues for each axis generated by the RDA indicate how much of the variation observed in species data can be explained by that axis. In this case, 91% of the correlation between experimental conditions, experiments and variables was explained by two axes (p-value = 0.03) (Fig. 5). For comparative purposes in this statistical study, data from experiments **1**–**8** were included together with data from experiments **9**–**11** (Table 1), which had an acidic initial pH or a slightly alkaline initial pH^9,18^. The first axis shows a positive correlation between the final pH and number of amino acids. The rest of the variables and experimental conditions showed a negative correlation with this axis, where the initial pH showed the highest negative correlation. The triplot showed a positive correlation between experiments performed with salts and aerosols and the total number of organic compounds identified, number of carboxylic acids and number of amino acids. The high pH experiments showed a positive correlation with the number of polyols, number of N-heterocycles and milligrams of hydrophilic *tholin*. Finally, the NH_3_-based atmosphere showed a positive correlation with the number of polyols, milligrams of hydrophilic *tholin*, milligrams of hydrophobic *tholin* and high final pH. The experiments were plotted on different areas of the diagram depending on their experimental characteristics. The combination of the first and second axes allowed discrimination among the different experiments. For example, experiments **7** and **8** were located in the negative part of the first axis and positive part of the second axis, because they were performed in the presence of salts, aerosols and NH_3_. Experiments **1**, **5** and **6** were plotted in the positive part of the fist axis and second axis because they were performed in the absence of salts. Experiments **2**, **9** and **10** were performed in the presence of aerosols and absence of NH_3_ and plotted in the positive part of the first axis and negative part of the second axis. Finally, experiments **3**, **4** and **11** were situated in the negative part of the first and second axes because they were performed in the presence of salts and aerosols. On the other hand, in general, the distribution of the experiments in the upper part or bottom part of the diagram is also related to the presence of NH_3_.

The correlations found with the initial pH are interesting. Experiments with an initial high pH plotted in the negative part of the first axis, and experiments performed at low pH plotted in the positive part of this axis, while the neutral initial pH values plotted the experiments in the central part of the graph. On the other hand, the experiments performed under extreme conditions (lowest pH and upper pH) presented a high correlation with the total number of organic compounds identified, especially if they were performed in the presence of aerosols and salts (experiments **4**, **8** and **9**). Between these samples, the special conditions of **4** (absence of NH_3_) provided more N-heterocycles, conditions of **8** (presence of NH_3_ and higher pH) provided more milligrams of hydrophilic *tholin* and conditions of **9** (lowest pH and absence of NH_3_) provided the highest number of amino acids.

In any case, independent of the presence of NH_3_, a greater diversity of organic compounds was related to the presence of aerosols, salts and alkaline pH values, as shown in Fig. 5. In addition, some advantages of alkaline pH values compared to acidic pH values for the emergence of life are that amino acids can be converted into peptides under alkaline conditions^26^ and alkaline pH values favour the polymerization and synthesis of possible protobiopolymers^9^. Although it is beyond the scope of the present paper, it is worth mentioning that the F_NH3_ fractions have a macromolecular nature with an electrophoretic mobility that is not present in the F_H2O_ fractions^9^ and that the presence of NH_3_ and alkaline pH values increases the production of these fractions and therefore the likely production of protobiopolymers. On the other hand, we propose that experiments with gas mixtures based on CH_4_, electric discharges, aqueous aerosols and alkaline pH values would lead to the production of sugars because the activation of CH_4_ with electric discharges leads to the formation of formaldehyde (e.g.^27^), aqueous aerosols increase the formation of hydroxylated compounds^8^, and the alkaline pH favours analogous reactions to result in the formose reaction (see e.g.^28^). In addition, it has been recently demonstrated that aerosols and alkaline pH might favour the emergence of a possible protometabolism when cyanide is used as the carbon source^12,15^, and it is well known that HCN is formed by the activation of CH_4_ and NH_3_/N_2_ by spark discharges^27^.

## Discussion

The chemical mixtures obtained in Miller-type experiments pose notable analytical challenges due to the heterogeneity of the samples and the large diversity of the obtained organic compounds. In this work, we focus on comparing several experimental conditions with respect to three main classes of polar organic compounds: carboxylic acids, N-heterocycles and amino acids. We also consider their possible implications for the main hypotheses about the origin life: i) the autotrophic origin, which can be related to the findings of some particular carboxylic acids, and ii) the heterotrophic origin, which can be related to the accumulation of the main components of nucleic acids and proteins.

### Carboxylic acids

**Figure 6:**
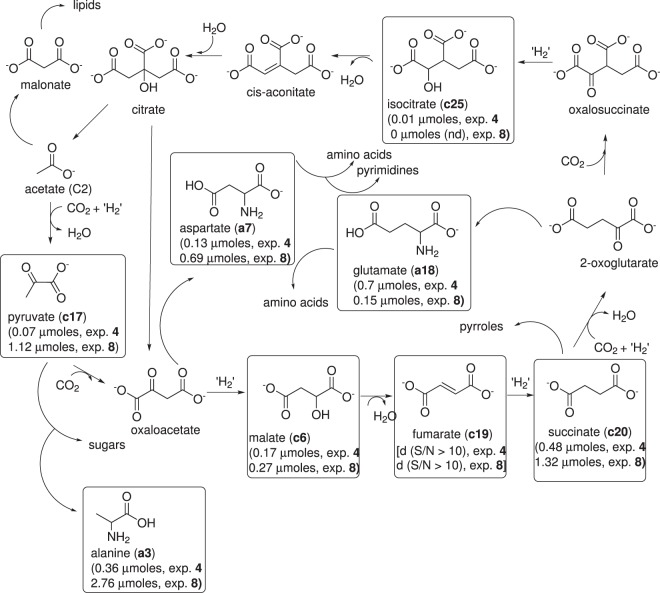
Reductive tricarboxylic acid cycle (rTCAC) (adapted from Guzman and Martin). In boxes the components of this cycle identified in hydrophilic *tholins* from experiments **4** and **8**, which used alkaline aqueous aerosols are marked. The µmoles for the carboxylic acids were calculated using the multiple point external standard method, and µmoles of amino acids were semi-quantitatively calculated from the peak of glycine, considering the related areas of the peaks versus the area of the peak of glycine. For the quantification of glycine, a multiple point external standard method was used.

The identification and formation of carboxylic acids under possible prebiotic conditions is a key component of the autotrophic hypothesis about the origin of life and the emergence of a primitive metabolism^29^. This hypothesis is mainly based on the reductive fixation of inorganic environmental carbon. In this context, the rTCA cycle has been proposed to be a plausible mechanism for carbon fixation and energy storage at the time of the emergence of life^29^. This cycle is the central axis of the universal metabolism. The combination of the rTCA cycle and its exit products operates as a factory for the synthesis of the main classes of biomolecules. This cycle is shown in Fig. 6. The key components identified via GC-MS in hydrophilic *tholins* from experiments **4** and **8** are marked in boxes. In general, quantitative/semiquantitative analyses of the carboxylic acids implicated in the rTCA cycles (Fig. 6) indicate that the yields for these compounds are increased when the plausible prebiotic conditions of experiment **8** are used. The rTCA cycle is effectively a mechanism of carbon fixation that can be started at any point along the cycle. Nonenzymatic chemical pathways for some steps of the rTCA cycle, similar to the initial input of the species involved, remain a challenging problem for the viability of the proposed prebiotic cycle. However, recently, some important advances have been made in this field^30–32^. In addition to those, herein, it is shown that alkaline reduced environments, especially in the presence of NH_3_, could be favourable scenarios for the emergence of some components of an ancient metabolism. However, the viability and likely emergence of the rTCA cycle in a non-enzymatic scenario has been evaluated^33^. In this sense, Zubarev and colleagues presented several examples of other auto-catalytic cycles in an rTCA supernetwork using a reactivity model^34^. These auto-catalytic cycles involve molecules with the highest closeness centrality in the rTCA network, and both sequences are based on glyoxylate (Fig. S6, Supporting Information). The glyoxylic acids (**c16**) and other compounds related to these cycles were found in the hydrophilic *tholin* from experiment **8** (Fig. S6, Supporting Information) in highly alkaline environments. Thus, some components of these auto-catalytic cycles could be available in a significant manner in alkaline environments. In addition, a protometabolic analogue of the rTCA cycle involving two linked cycles has recently been experimentally demonstrated^35^. In this approach, to understand the origin of life, glyoxylate acts as a carbon source, and pyruvate is used as one main reagent. On the other hand, in the abovementioned “glyoxylate scenario”, glyoxylate and its formal dimer, dihydroxyfumarate, are suggested to be the key starting materials of the chemical constitution of a possible metabolism, serving as a source of the main biomonomers, such as sugars, amino acids, pyrimidines and the constituents of the rTCA cycle^25^. To the best of our knowledge, few works describe plausible prebiotic syntheses of glyoxylate^12,36,37^. Thus, the alkaline conditions using aqueous aerosols described here can be additional prebiotic sources for this important keto acid due to the relatively high yields obtained.

### N-Heterocycles

The hypothesis of an RNA world, wherein RNA plays both the role of an informational carrier and the role of a catalyst, is one of the most popular early life hypotheses. In contrast, the emergence of RNA from potentially natural pathways is still open to debate, and it is postulated that RNA could be a product of a multistep evolution process, as proposed in^38^. In this line of thinking, the development of non-natural base pairs for incorporation into DNA and RNA has been the focus of several research groups for a number of years, e.g.^39^. Recent studies investigating RNA as an early genetic code found that cyanuric acid (**h13**) and triaminopyrimidine self-assemble in water to create aggregates resembling contemporary nucleic acid base pairs. Triaminopyrimidine forms nucleosides with ribose, and upon heating, the cyanuric acid (**h13**) mixture forms gene-length polymers that have been termed proto-RNA^40^. This polymerization suggests that cyanuric acid (**h13**) and related cyclic compounds may have played a role in the initiation of life on Earth. To test this hypothesis, prebiotic production of cyanuric acid (**h13**) must be demonstrated. Here, prebiotic synthesis of cyanuric acid (**h13**) is demonstrated, indicating that its production is possible using alkaline aqueous aerosols, a reductive atmosphere and spark discharges. This synthetic method increases the scarce number of possible prebiotic syntheses previously reported: from the hydrolysis of HCN polymers^12^; from CO, H_2_, and an NH_3_ mixture^41^; and from urea subjected to freeze-thaw cycles and spark discharges under CH_4_/N_2_/H_2_ or argon^6^. In the last case, the formation pathway of cyanuric acid (**h13**) is based on the fact that biuret (NH_2_CONHCONH_2_) is a condensation product of two urea molecules and is a precursor of cyanuric acid (**h13**). The use of spark discharges for the formation of cyanuric acid (**h13**) suggests that free radicals are involved in the processes^42^. In the present case, the production of cyanuric acid (**h13**) would follow the same pathway because urea was formed in all of the experiments analysed (**1**–**8**), and iminoacetic acid (**a12**), the oxidase form of the biuret, was also identified. Moreover, it is well known that aqueous aerosols stabilize and favour the production of free radicals, e.g^18^. On the other hand, ribonucleotides and RNA are also thought to be preceded by simpler progenitors comprising the nucleotides containing C3 and C4 units based on glycerol and tetrose sugar as information carriers. Flexible nucleic acid (FNA)^43^, glycerol nucleic acid (GNA)^44^, and isoGNA^45^, based on a simple 3-carbon glycerol, were thus considered. In this manner, the identification of glycerol (**o1**) in the hydrophilic *tholins* from experiment **8** is also very interesting because this is the first time that this compound has been found under simulating spark discharge conditions and because the prebiotic syntheses of these biological compounds have rarely been described^46–48^. In addition, beyond its role as a constituent of a pre-RNA world, glycerol (**o1**) is well known to be a key building block of lipids, which are central components of all cellular membranes. Additionally, acyclic nucleic acid based on glyceric acid (**c5**) has been proposed^49^.

### Amino acids

The identification and quantification/semi-quantification of glycine (**a1**), alanine (**a3**), and aspartic (**a11**) and glutamic acids (**a18**), in addition to those of glycolic (**c1**), lactic (**c2**), malic (**c6**) and 2-hydroxyglutaric (**c13**) acids, are in good agreement with the findings of Parker and colleagues based on analyses of several Miller-type reaction mixtures via ultrahigh-performance liquid chromatography and triple quadrupole mass spectrometry^50^. These results indicate that the production of depsipeptides as a plausible prior step to the prebiotic formation of polypeptides^51^ could be initiated using glycine (**a1**), alanine (**a3**), and glycolic (**c1**) and lactic (**c2**) acids due to the presence of major compounds in the mixture reactions, along with smaller quantities of aspartic (**a11**), glutamic (**a18**), malic (**c6**) and 2-hydroxyglutaric (**c13**) acids. Our detection limits using GC-MS are greater than those reported by Parker *et al*^50^. when using UHPLC-QqQMS. Additionally, our yields are also minor, probably due to the work-up and derivatization of the samples since in the work of Parker *et al*., the samples are directly injected in solution onto the chromatography column. In any case, the ratio between the different amino acids and hydroxy acids is similar, and the production of these compounds is favoured in an alkaline medium, as in the case of Parker *et al*.^50^. On the other hand, in relationship with the abiotic production of peptides, hydantoins have been suggested to be a precursor for the emergence of prebiotic peptides and amino acids. Moreover, it has been hypothesized that primitive microorganisms on Earth may be able to use hydantoins as C or N sources. The findings presented here demonstrate that hydantoins can be prebiotically synthesized using reduced gas mixtures activated by spark discharges at middle temperatures. These synthetic conditions extend the scope of experiments that lead to the abiotic synthesis of hydantoins, which was previously always demonstrated in icy environments^6,37,52^.

Therefore, the analyses of the *tholins* here described show that some components implicated in an autotrophic origin of life and those implicated in a heterotrophic origin can be synthetized simultaneously under particular environmental conditions: alkaline aerosols, reduced gas mixtures and an external energy source. The existence of alkaline lakes on the modern Earth, and their plausible existence on the early Earth, increases the likelihood of auspicious alkaline environments that are capable of generating bioorganics. Finally, alkaline aerosols and a reduced atmosphere may have been possible on ancient Mars due to the past volcanic activity of this planet and the recently demonstrated existence of ancient alkaline lakes, such as that located at Gale Crater, where the Curiosity Rover is operating^53^. In addition, although not discussed in this work, a protocell can also be defined as a large ordered structure enclosed by a membrane that performs some life activities, such as growth and division. These last characteristics can be found in atmospheric aerosols^54^. Thus, plausible alkaline environmental conditions and the analytical findings checked for the *tholins* synthetized under such conditions lead to proposing a vision about the origin of life such as that previously suggested by Eschemnoser via “an iconoclastic attitude among genetic, metabolism, and compartmentalization towards one of openness to horizontal transfer of ideas and insights” in the field of the prebiotic chemistry^55^.

## Conclusions

It has been speculated that a non-enzymatic version of the rTCA cycle may have been central to the origin of life. Our experiments demonstrate both the formation of key elements of the rTCA cycle and of some sugar precursors. In particular, our experiments demonstrate that salinity and pH significantly change the nature of both hydrophilic and hydrophobic *tholins*. This is the first time that an α-ketoacid that forms part of the rTCA cycle has been detected in *tholins* from spark discharge experiments. Quantification of the pyruvic acid in *tholins* shows yields based on a carbon fixation of up to 0.044%. In general, the yields increase with the increase of the initial pH for the experiments of the CH_4_ + NH_3_ + H_2_ series, and the yield for the experiments at an initial pH 12 is greater with NH_3_. Glyoxylic acid was found with a yield of 0.078%. It is also notable that glycerol was identified to have a yield of 0.238%. This is the first time that a sugar derivative has been found in this type of prebiotic simulation. Indeed, contrasting our analysis with the control experiments demonstrates that the presence of aerosols, salts and alkaline pH values leads to a greater diversity of organic compounds. The volcanic scenario proposed here not only allows for the simultaneous emergence of metabolic, informational and structural components of a plausible primitive biochemistry but also the availability of containers for them. Our experiments confirm that volcanic environments next to alkaline pools of water would be ideal prebiotic niches for the accumulation of organics with biological interest.

## Electronic supplementary material

Supplementary Information
