# Supplementary material for: Prebiotic chemistry in neutral/reduced-alkaline gas-liquid interfaces
Source: Sci Rep. 2019 Feb 13;9:1916. doi: 10.1038/s41598-018-36579-7 (PMC6374446; doi:10.1038/s41598-018-36579-7)
Supplement: Supplementary file 1 — Supplementary Information [file 41598_2018_36579_MOESM1_ESM.pdf]

Prebiotic chemistry in neutral/reduced-alkaline gas-liquid interfaces

Cristina Mompeán, Margarita R. Marín-Yaseli, Patricia Espigares, Elena González-Toril, M.-Paz Zorzano, Marta Ruiz-Bermejo *

Supporting Information

Materials and Methods

Simulation of alkaline reductive environments

A 500 mL glass reactor was filled with a gas mixture containing CH_4:NH_3:H_2 (40:30:30), purchased from Air Liquid, and 5 mL of a saline solution 1.5 times the concentration of sea water [0.63 M NaCl, 0.18 M $MgCl_2$, 0.015 M KCl, 0.02 M $NaHCO_3$ (Ruiz-Bermejo et al. 2007a and 2011) adjusted to various pH levels: pH = 9.8, with $Mg(OH)_2$ or pH = 12 with NaOH (experiments **7** and **8**, Table 1). In addition, two control experiments were performed with pure water. One of these experiments used liquid water at 38 ºC (experiment **5**, Table 1), and the second used aqueous aerosols (experiment **6**, Table 1). NaCl, $MgCl_2$, $CaCl_2$ and KCl as well as NaOH and HCl (6N) were obtained from Panreac, and $NaHCO_3$ and Mg $(OH)_2$ were obtained from Fluka. The solutions or suspensions were bubbled with dried N_2 for 1 h, and prior to use, all glassware and electrodes were heated in a high-temperature oven (Nabertherm Labotherm L5) at 400 ºC in air for 2 h to eliminate any possible organic contaminants. Before each experiment, the system was evacuated with a membrane pump and purged with the reaction gas mixture 4 times. After this process, the reactor was filled until a pressure of 1,200 mbar was reached. An ultrasonic aerosol generator (BONECO model 7035) working at 1.8 MHz and 33 W was used for the bubble-sol cycle to generate aqueous aerosols or alkaline saline aerosols in a few minutes (see, e.g., Ruiz-Bermejo et al. 2007b). Two tungsten electrodes attached to the reactor were used with a high voltage generator (Model BD-50E, Electrotechnic Products, Inc., Illinois, USA) to produce spark discharges (50 kV). The system was maintained at a constant temperature (38 ºC) with active aerosol and electric discharge for 72 hours. After this period, the liquid solutions and solid materials were separated by centrifugation. The supernatants (hydrophilic t*holins*) and precipitated solids (hydrophobic *tholins*) were freeze-dried and recovered for analytical and spectroscopic studies.

Instrumental Analyses

Elemental analysis: Hydrophilic and hydrophobic *tholins* were examined for the determination of the mass fractions of carbon, hydrogen and nitrogen using a PerkinElmer elemental analyser, model CNHS-2400. The % oxygen of the samples was calculated by difference.

Infrared Spectroscopy: IR spectra were obtained using a Nexus Nicolet FTIR spectrometer. The spectra were obtained in CsI pellets using the reflectance mode of operation.

FT-IR spectra of *tholins* from pure water experiments (Experiments **1**-**2** and **5**-**6**)

Because NH_3 is more reactive than N_2, structural differences were expected between the *tholins* synthesized from CH_4+N_2+H_2 with respect to those produced from CH_4+NH_3+H_2. Figure S1 shows the FT-IR spectra of the hydrophilic and hydrophobic *tholins* obtained using pure water and a CH_4+NH_3+H_2 atmosphere (experiments **5** and **6**). These FT-IR spectra are similar to those reported previously for the *tholins* synthesized from CH_4+N_2+H_2 (experiments **1** and **2**) (Ruiz-Bermejo et al. 2007b, 2008 and 2009. Additionally, see these references for a comprehensive assignment of the features). The main difference found between the hydrophilic *tholins* obtained from these two atmospheres is that the band related to the nitrile groups appears clearly unfolded in the experiments performed using NH_3, with a new shape at 2188 cm^{-1} (Figure S1a). These bands can be associated with nitrile conjugated to C = C when a strong electron-donating group, such as an amino moiety (-NH_2 or -NR_2), is present on the β-carbon on the double bond. In the case of the hydrophobic *tholins* from CH_4+N_2+H_2 (Ruiz-Bermejo et al. 2007a and 2009), the band related to nitriles appears unfolded at ~ 2235 and ~ 2210 cm^{-1}, while in the case of hydrophobic *tholins* from CH_4+NH_3+H_2, these unfolded bands are found at ~ 2235 and ~ 2190 cm^{-1} (Figure S1b). Additionally, a well-defined band is found at 1529 cm^{-1} in the hydrophobic *tholins* from experiments **1** and **2** (Ruiz-Bermejo et al. 2007b). However, this band appears as a shoulder for the hydrophobic *tholins* from experiments **5** and **6**. Moreover, the relative intensity of the band related to the nitrile groups with respect to the band related to alkane groups is greater in the hydrophobic *tholins* from experiments **1** and **2** than in the hydrophobic *tholins* from experiments **5** and **6**.

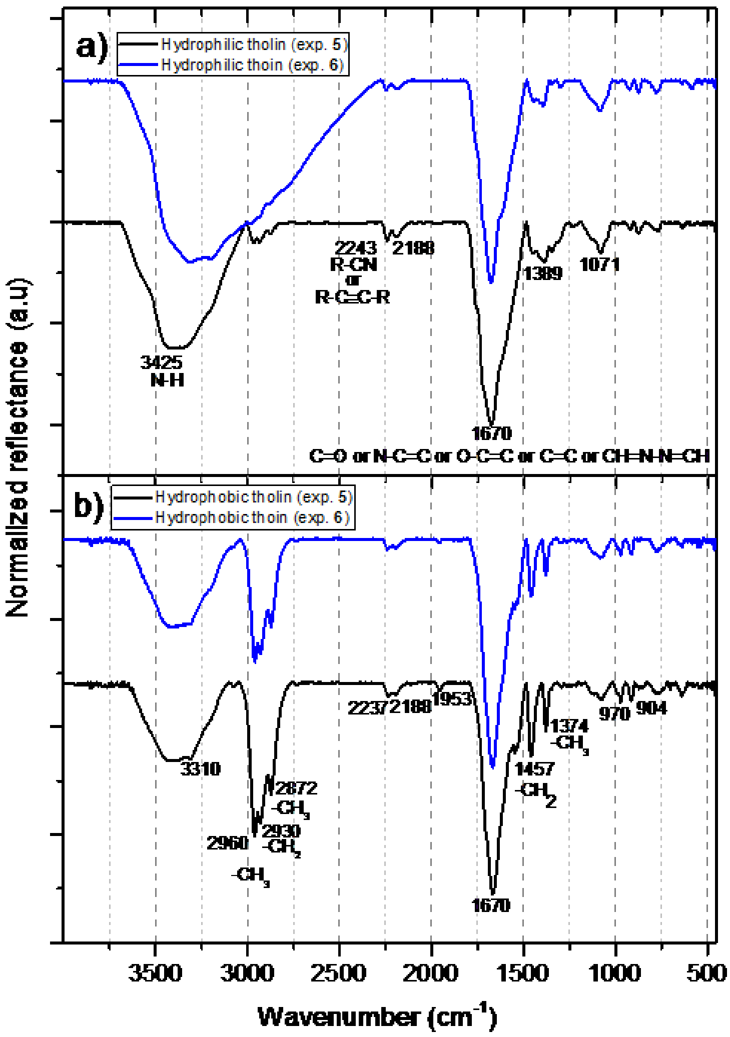

***Figure S1**. FT-IR spectra of tholins produced using pure water and an atmosphere of CH_4 + NH_3 + H_2. a) Hydrophilic tholins; b) Hydrophobic tholins.*

Elemental analysis of *tholins* from pure water experiments (Experiments **1**-**2** and **5**-**6**)

From the data from the elemental analysis results, structural differences can be observed between the hydrophilic and hydrophobic *tholins* from experiments **1** and **2** and from experiments **5** and **6** (Figure 2S). The hydrophilic *tholins* from a CH_4 + NH_3+ H_2 atmosphere (experiments **5** and **6**) present a greater functionality than the hydrophilic *tholins* from CH_4 + N_2 + H_2 (**1** and **2**), especially in the case of the aqueous aerosols experiments (Figures 2Sa, 2Sb and 2Sc). The result is the same for the case of the hydrophobic tholins (Figures 2Sd, 2Se and 2Sf), i.e., although the hydrophobic *tholins* have a very different nature than the hydrophilic tholins, as observed above by FT-IR spectroscopy and in previous works (Ruiz-Bermejo et al. 2008 and 2009), the hydrophobic tholins obtained from an atmosphere containing NH_3 present a greater amount of nitrogenized and oxygenated functional groups than the corresponding hydrophobic *tholins* produced from N_2, and moreover, the presence of aqueous aerosols increases the diversity of functional groups.

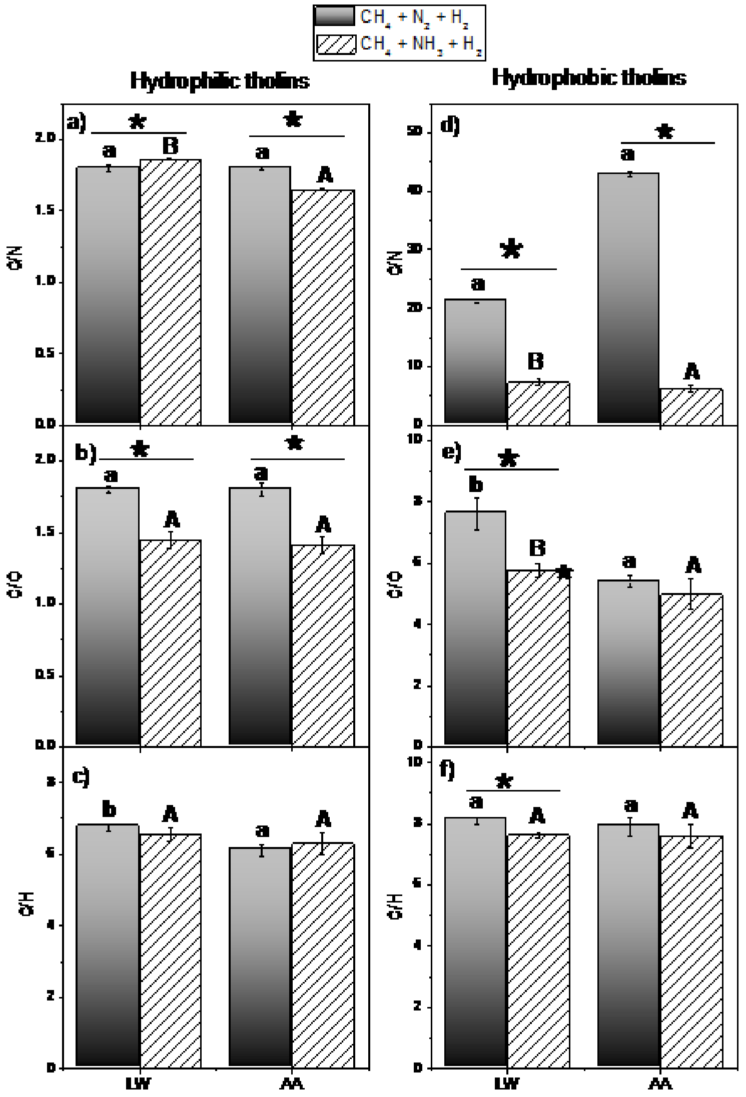

Figure S2*. Histograms showing elemental analyses data for hydrophilic and hydrophobic tholins: a) Relationship of C/N for hydrophilic tholins; b) relationship of C/O for hydrophilic tholins; c) relationship of C/H for hydrophilic tholins; d) relationship of C/N for hydrophobic tholins; e) relationship of C/O for hydrophobic tholins; f) relationship of C/H for hydrophobic tholins. LW = spark discharges experiments using liquid water (pure water, initial pH = 7, experiments* ***1*** *and* ***5****); AA = spark discharges experiments using aqueous aerosols (pure water, initial pH = 7, experiments* ***2*** *and* ***6****). Statistical test: pairs test bases on confident interval comparison (α < 0.05). * indicates differences within groups, and letters indicate differences between groups (CH_4+N_2+H_2 atmosphere group = lowercase letters; CH_4+NH_3+H_2 atmosphere group = capital letters).*

Structural comparative between *tholins*: Influence of salinity and pH

The notable variations observed in the relationship of F_{N3H}/F_{H2O} indicate that the nature of the hydrophilic *tholins* produced using NH_3 is very different than those produced without. Thus, in Figures S3a and S3b, one can see that the FT-IR spectra of the F_{NH3} fractions from experiments **3** and **4** and from experiments **7** and **8** do not maintain any similarity between them. This is not the case for the hydrophilic *tholins* obtained from pure water using different sources of nitrogen because the differences found between the FT-IR spectra were minimal, as mentioned above (please compare Figure S1 with Figures S3).

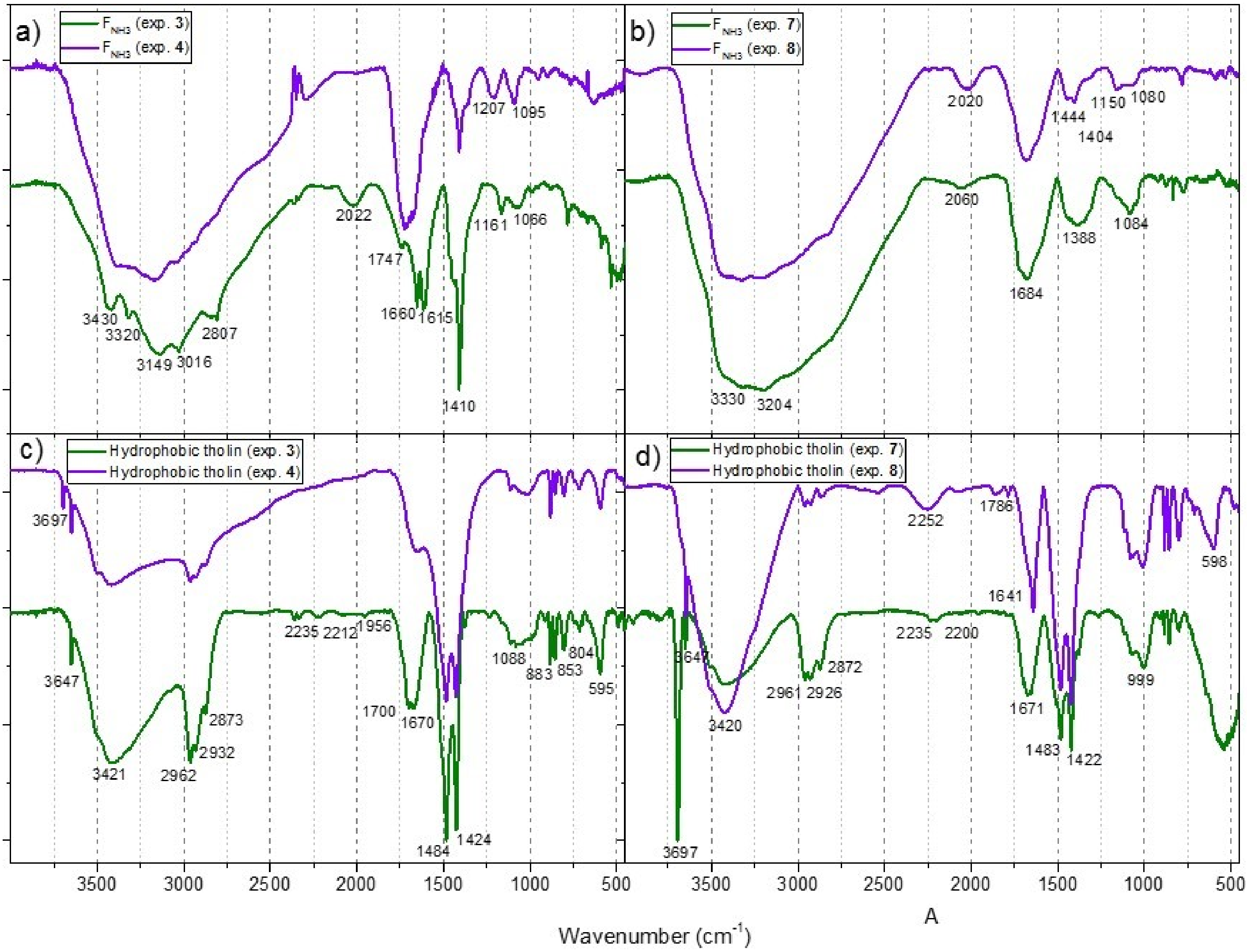

***Figure S3**. FT-IR spectra of a) F_{NH3} fractions from hydrophilic tholins synthesized using alkaline aqueous aerosols with different initial pH values and an atmosphere of CH_4 + N_2 + H_2; b) F_{NH3} fractions from hydrophilic tholins synthesized using alkaline aqueous aerosols with different initial pH values and an atmosphere of CH_4 + NH_3 + H_2; c) hydrophobic tholins from an atmosphere of CH_4 + N_2 + H_2 and alkaline aqueous aerosols; and d) hydrophobic tholins from an atmosphere of CH_4 + NH_3 + H_2 and alkaline aqueous aerosols.*

Therefore, together with the differences produced by the nitrogen source, salinity and pH, the above result leads to significant variations in the nature of the final water soluble products. For example, the relationships of C/N, C/O and C/H for the F_{NH3} from experiments **7** and **8** are very different (Figure S4) with respect to those found for the hydrophilic *tholin* from experiment **6** synthetized using pure water and aqueous aerosols (Figure S2). The F_{NH3} from experiments **7**

and **8** have more functional groups containing nitrogen and oxygen than the corresponding hydrophilic *tholin* from experiment **6**. The relationship shown in Figure S4 also indicates significant structural differences between the F_{NH3} fraction obtained from solutions/suspensions at different initial pH values. The F_{NH3} fraction from experiment **7** is more highly functionalized than the corresponding fraction from experiment **8**.

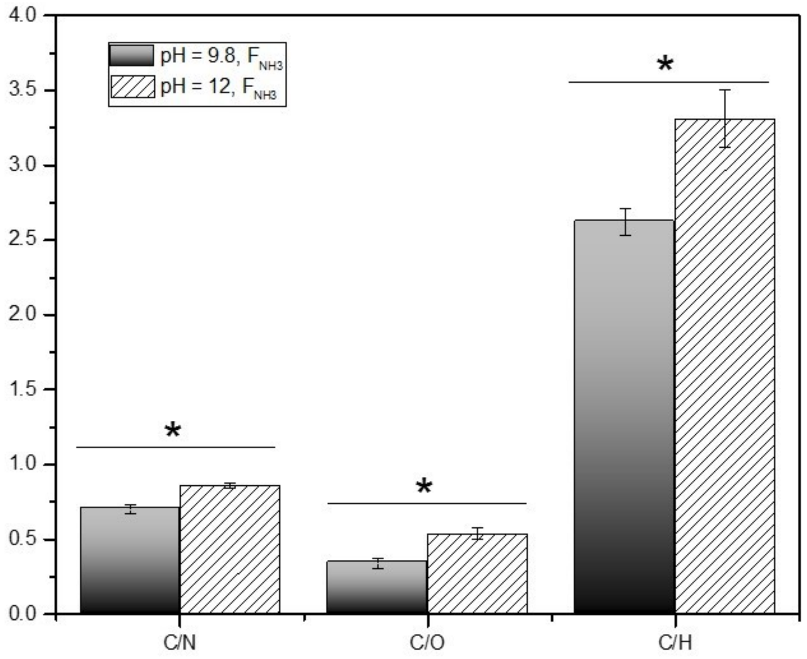

Figure S4. *Elemental analysis data for the ammoniacal fractions, F_{NH3}, from hydrophilic tholins obtained in the experiments using alkaline aqueous aerosols and a CH_4 + NH_3 + H_2 atmosphere.*

For the hydrophobic *tholins* synthesized using alkaline aqueous aerosols, similar conclusions can be obtained to those indicated from the hydrophilic *tholins* and F_{NH3} fractions from the corresponding data of the FT-IR spectra. While the effect of the nitrogen source seems to lead to minor changes in the FT-IR spectra of the hydrophobic *tholins* synthesized using pure water, i.e., experiments **1**-**2** and **5**-**6** (Ruiz-Bermejo et al. 2007b and Figure S1b), the variations in the salinity and pH substantially modify aspects of the FT-IR spectra (Figures S3).

Semiquantitative analysis of the main polar compounds identified in hydrophilic tholins (Experiments **4** and **8)**

Table S1 shows the semiquantitative analyses of experiments **4** and **8** regarding the main compounds for each group of polar compounds analyzed by GC-MS in the present work (amino acids, carboxylic acids and N-heterocycles).

Table S1. *GC-MS semiquantitative analysis of the mayor compounds found in the F_{NH3} fractions from experiments **4** and **8** for the three groups of the main polar compounds studied here: amino acids, carboxylic acids and N-heterocycles. The data are indicated in µmol of the compound. The semiquantitative analyses were performed using glycine as an internal standard. The quantification of glycine (**a1**) was achieved using the multiple point external standard method, using standard solutions from 10 ppm to 100 ppm. For the semiquantification of the compounds shown in this table, the µmols found in the F_{NH3} and F_{H2O} fractions were combined.*

**Compound**	Exp. **4**	Exp. **8**
**AMINO ACIDS**		
Glycine	2.90	16.37
Alanine	0.36	2.76
β-alanine	0.26	1.05
**CARBOXYLIC ACIDS**		
Glycolic acid	1.91	2.99
Oxalic acid	1.34	5.01
2-Methylsuccinic acid	0.06	2.21
Succinic acid	0.48	1.32
**N-HETEROCYCLES**		
5-hydroxyhidatoin	0.17	0.59
Orotic acid	0.03	0.19
Cyanuric acid	0.25	0.006

Analyses of non-hydrolyzed samples and the subfractionations of the F_{NH3} fractions

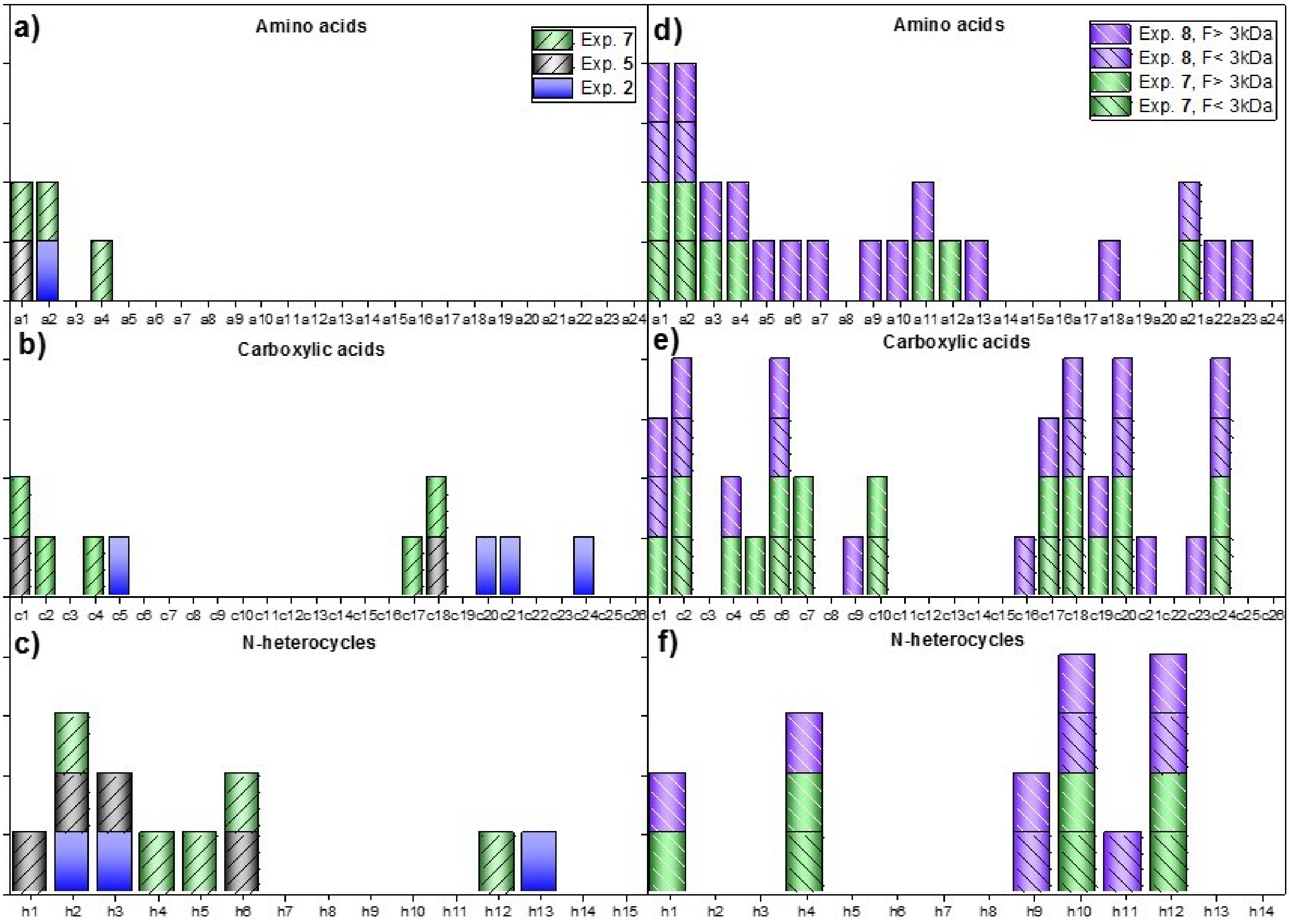

Figure S5. *Organic polar compounds identified as their TMS-derivatives by GC-MS in non-hydrolysed samples: the bulk hydrophilic tholins from experiments **2** and **5** and the F_{NH3} fraction from experiment **7**, obtained by the desalting of the corresponding hydrophilic tholin (Figures a, b and c); and organic polar compounds identified as their TMS-derivatives by GC-MS in acid hydrolysed subfractions collected using centrifugal filter devices with a cut-off of 3 kDa [light fraction (< 3 kDa) and a heavy fraction (> 3 kDa)] from the F_{NH3} fractions, obtained by the desalting of hydrophilic tholins synthesized in the presence of alkaline aqueous aerosols at an initial pH = 9.8 and 12 (experiments **7** and **8**, respectively) (Figures d, e and f). The numbers shown under the abscissa axis correspond with the numeration of analytes shown in Figure 2. The coloured bars indicate the presence of a concrete analyte in the corresponding sample.*

Auto-catalytic cycles in an rTCA supernetwork

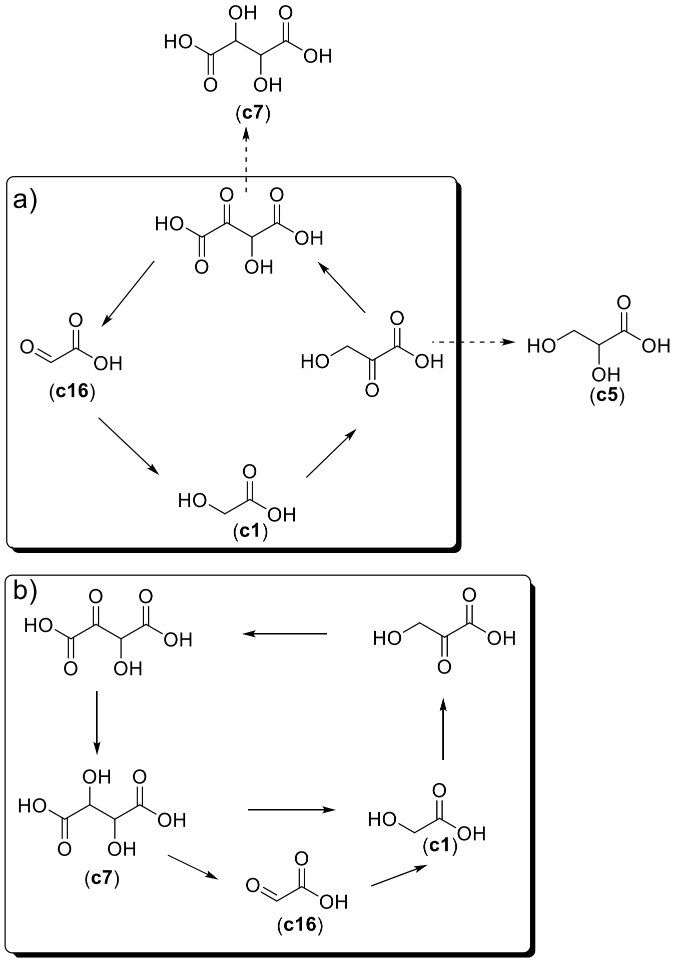

Figure S6. *Autocatalytic cycles found in the rTCA supernetwork are represented as boxes (Zubarev et al. 2015). The numbered compounds were identified by GC-MS in tholins produced using alkaline aqueous aerosols and a reductive atmosphere.*

References

Ruiz-Bermejo, M., Menor-Salván, C., Osuna-Esteban, S., Veintemillas-Verdaguer, S. The effects of ferrous and other ions on the abiotic formation of biomolecules using aqueous aerosols and spark discharges. *Origin Life Evol Biosph.* **37**, 507-521 (2007a).

Ruiz-Bermejo, M.; Menor-Salván, C., Osuna-Esteban, S.; Veintemillas-Verdaguer, S. Prebiotic Microreactors: A Synthesis of Purines and Dihydroxy Compounds in Aqueous Aerosol. *Origin Life Evol B*, **37**, 123-142 (2007b).

Ruiz-Bermejo, M.; Menor-Salván C.; Mateo-Martí, E.; Osuna-Esteban, S.; de la Fuente, J. L.; Martín-Gago, J. A.; Veintemillas-Verdaguer, S. $CH_4/N_2/H_2$ Spark hydrophobic *tholins*: A systematic approach to the characterization of tholins. Part II. Icarus ***204,*** 672-680 (2009).

Ruiz-Bermejo, M.; Menor-Salván C.; Mateo-Martí, E.; Osuna-Esteban, S.; Martín-Gago, J. A.; Veintemillas-Verdaguer, S. $CH_4/N_2/H_2$ Spark hydrophilic tholins: A systematic approach to the characterization of tholins. Icarus **198**, 232-241 (2008).

Zubarev, D. Y., Rappoport, D., Aspuru-Guzik, A. Uncertainty of prebiotic scenarios: The case of the non-enzymatic reverse tricarboxylic acid cycle. *Scientific Reports* **5**, 8009 (2015).
